# Diagnosis and prevalence of ovine pulmonary adenocarcinoma in lung tissues of naturally infected farm sheep

**DOI:** 10.14202/vetworld.2016.365-370

**Published:** 2016-04-11

**Authors:** Ganesh G. Sonawane, Bhupendra Nath Tripathi, Rajiv Kumar, Jyoti Kumar

**Affiliations:** 1 Animal Health Division, ICAR-Central Sheep and Wool Research Institute, Avikanagar, Malpura, Tonk, Rajasthan, India; 2 ICAR-National Research Centre on Equines, Hisar, Haryana, India; 3 Animal Biotechnology Section, ICAR-Central Sheep and Wool Research Institute, Avikanagar, Malpura, Tonk, Rajasthan, India; 4 Animal Health Division, ICAR-Central Sheep and Wool Research Institute, Avikanagar, Malpura, Tonk, Rajasthan, India

**Keywords:** diagnosis, lung tissues, ovine pulmonary adenomatosis, sheep

## Abstract

**Aim:**

This study was aimed to detect ovine pulmonary adenocarcinoma (OPA) in sheep flocks affected with pulmonary disorders at organized farm.

**Materials and Methods:**

A total of 75 sheep died naturally were thoroughly examined for the lesions of OPA during necropsy. Tissue sections from affected portion of the lungs from each animal were collected aseptically and divided into two parts; one each for polymerase chain reaction (PCR) and another for histopathology.

**Results:**

On PCR examination of lung tissues, six sheep (8%) were found to be positive for JSRV. Two of them were 3-6 months of age and did not show clinical signs/gross lesions of OPA. Four adult sheep positive on PCR revealed characteristic lesions of OPA on gross and histopathological examination.

**Conclusion:**

In the absence of known specific antibody response to the infection with JSRV, there is no diagnostic serological test available. The PCR assay employed in this study on lung tissues, using primers based on the U3 region of the viral long terminal repeat for JSRV would be helpful in the screening of preclinical and clinical cases of OPA in sheep.

## Introduction

In Rajasthan sheep and goats are generally reared through an extensive system of rearing and are one of the major sources of sustainable livelihood of rural poor and have great economic value in terms of meat, wool and milk. In the present scenario, the demand for meat in India has increased rapidly, and emphasis has shifted from wool toward mutton as the main produce from sheep rearing [1]. Respiratory infections are quite common and responsible for 30-40% of mortality in sheep of various ages. Ovine pulmonary adenocarcinoma (OPA) has been identified in a wide variety of breeds in many countries around the world including India [2]. It is responsible for severe economic losses to the sheep industry in many sheep rearing countries and the subclinical form of the disease affects growth rate, carcass weight, and milk and wool production [3]. OPA is believed to be the most important disease that can affect international trade as determined by the Office International des Epizooties (OIE) [4]. In addition to its importance as a veterinary problem, OPA has widerrelevance for fundamental studies on cancer because it has similar pathological and epidemiological features to bronchoalveolar carcinoma in humans, and is considered a useful animal model of pulmonary carcinogenesis. OPA is a contagious lung cancer of sheep previously known as sheep pulmonary adenomatosis and ovine pulmonary carcinoma [5]. OPA is caused by Jaagsiekte sheep retrovirus (JSRV), which induces oncogenictransformation of alveolar and bronchiolar secretory epithelial cells. In addition to sheep, goats and wild moufflon are also susceptible to the virus [6]. Natural cases of OPA occur in sheep at 2-4 years of age; the disease may affect lambs at the age of 2 months [7]. The incubation period in naturally infected animals is reported to be 6 months to 3 years. The clinical signs are progressive emaciation, weight loss and respiratory distress, particularly after exercise. Affected sheep often lag behind the flock. There is usually a thin mucus discharge from the nostrils, and if the head is lowered, a copious frothy exudate may pour from the nares. Moist rales may be heard on auscultation, but coughing is not usually prominent. The clinical signs are slowly progressive, ending in severe dyspnea. Death usually occurs in days to a few months, often from secondary bacterial pneumonia [7-9].

OPA can be transmitted via aerosols or droplets. The horizontal transmission has been demonstrated among sheep of all ages, but neonates seem to be particularly susceptible to infection. JSRV can be found in tumors, lung fluids, peripheral blood leukocytes and lymphoid organs and before the development of tumor, the virus can be detected in lymphoreticular cells [10]. There is no evidence that *in utero* transmission is statistically significant in the epidemiology of this disease; however, recent studies suggest that JSRV might be spread through milk or colostrum. JSRV does not survive for long periods in the environment [11].

Diagnosis of OPA is possible when clinical signs or tumors are observed [12], and the presence of JSRV can be confirmed in lung fluid or tumors by immunoblotting [13], ELISA [14] or polymerase chain reaction (PCR) [15-17]. However, it is more difficult to identify infected animals during the pre-clinical period due to the lack of detectable JSRV proteins outside the tumor [14] and of circulating JSRV-specific antibodies [18]. However, no routine assays for preclinical diagnosis of JSRV infection are available. In India, very few studies have been conducted for the detection of OPA and limited to the histopathological diagnosis only. OIE has mentioned PCR assays for the detection of JSRV in blood, bronchoalveolar lavage, lung, and lymph node tissues; however, we face difficulty in amplification of targeted gene from the lungs showing gross and histopathological OPA lesions. Therefore, the protocol for DNA extraction and PCR assay was slightly modified and optimized. In the absence of serological test and cell culture system for JSRV isolation, there is no confirmatory laboratory method for the antemortem diagnosis of OPA in affected animals and primary diagnosis can be made on the basis of flock history, clinical signs, and post-mortem lesions. The disease can be confirmed by histopathology and PCR examination. In our study, the aim was to confirm the existence and prevalence of OPA in naturally died animals exhibiting gross lesions in lungs related to OPA, therefore, histopathology and PCR used.

## Materials and Methods

### Ethical approval

Ethical approval was not necessary as samples were collected from dead animals.

### Animals and necropsy

In this study, a total of 75 sheep died naturally were thoroughly examined for the pneumonic and OPA lesions during necropsy. The history was collected from the records of the farm under study and postmortem requisition forms received in the division. Detailed history on clinical signs including body weights, progressive emaciation, etc., was recorded before necropsy examination. Tissue sections from affected portion of the lungs from each animal were collected aseptically and divided into two parts; one each for PCR and another for histopathology. All tissues to be used for DNA extraction was transported to laboratory on ice and stored at −20°C until further use. For histopathology, tissues were preserved in 10% neutral buffered formalin.

### Histopathology

The formalin fixed tissues were cut into pieces of 2-3 mm thickness and washed thoroughly with water for several hours before putting in ascending grades of alcohol for dehydration. The dehydrated tissues were cleared in xylene and embedded with paraffin. Sections of 4-5 µm thickness were prepared from paraffin blocks and stained with hematoxylin and eosin [19].

### DNA extraction and PCR

The genomic DNA was isolated from the lungs of 75 sheep using commercial DNA extraction kit (Himedia, India) as per the method described by the manufacturer. The primers (Jag F, 5’-TGGGAGCTCTTTGGCAAAAGCC-3’, Jag R, 5’-CACCGGATTTTTACACAATCACCGG-3’) flanking a region of 176 bp region of U3-LTR gene [10] and (F,- 5’TGTTCCAGTATGATTCCACCC-3’; R, 5’-ATAAGTCCCTCCACGATGCC-3’) product size 388 bp, specific to ovine glyceraldehyde-3-phosphate dehydrogenase (GAPDH) gene [20] were synthesized commercially (Sigma–Aldrich) and used. The samples that were negative by the U3-PCR were tested by PCR for GAPDH to verify the integrity of the DNA [10].

The genomic DNA isolated from tissues were amplified in 50 µl reaction mixture containing 1× PCR buffer, 1.5 mM MgCl_2_, 200 mM of dNTPs, 0.5 U of Taq polymerase (MBI Fermantas, MO, USA), 1 µM of primers (Jag F and Jag R), and 2 µl (0.6-0.8 µg) of purified genomic DNA solution. The PCR conditions consisted of initial denaturation at 94°C for 10 min, 40 cycles each of denaturation at 94°C for 1 min, annealing at 59°C for 1 min and synthesis at 72°C for 1 min, and final elongation at 72°C for 3 min. In every batch of PCR, negative (DNA from lungs tissues of healthy sheep amplified with ovine specific GAPDH primers) control was included. The PCR products were analyzed by visualization of desired size of DNA band in the ethidium bromide-stained agarose gel (2.0%). Unfortunately positive virus nucleic acids are not available with us, however, confirmed OPA positive animals (Based on gross and histopathology results) were considered as a positive control to screen other samples suspected to be positive for OPA.

## Results

### PCR examination

**Figure 1:**
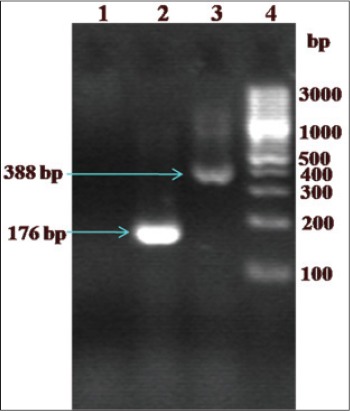
Polymerase chain reaction (PCR) amplification of U3 gene fragment of Jaagsiekte resolved on 2.0% agarose gel electrophoresis: Lane 1 - Non-template PCR control; Lane 2 - Amplification of JAG U3 gene; Lane 3 - Negative control (with healthy sheep lungs DNA and glyceraldehyde-3-phosphate dehydrogenase primers) and Lane 4: 100 bp plus DNA ladder (Fermentas, Cat SM0323).

A total of 75 sheep died naturally were thoroughly examined for the lesions of OPA during necropsy. On PCR examination of lung tissues using U3-LTR gene, of 75, six (8%) sheep (four adult and two lambs) were found to be positive for JSRV (Figure-1). In rest of the animals, macroscopic and microscopic findings in the lungs were related to various types of pneumonia (congestion/hemorrhage, edema, consolidation and suppuration, etc.) and considered as other than OPA lesions. We feel it will be unrelated to mention findings of the each and every case in the present manuscript. GAPDH PCR product length in negative animals was same as in positive animals as expected.

### Gross lesions

**Figure 2:**
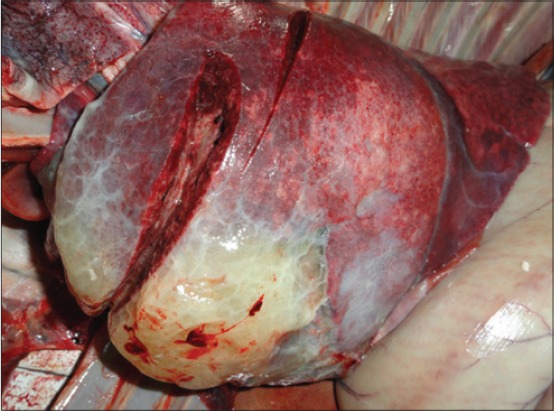
Ovine pulmonary adenocarcinoma affected sheep lung showing pearly-white, hard nodule.

The adult PCR positive sheep revealed characteristic lesions of OPA on gross and histopathological examination. Clinical history of these animals revealed weakness, anorexia, panting, cough, and sneezing. Grossly, the lungs appeared grayish to light purple in color, which were enlarged in size and heavier. Consistency varied from granular to meaty. On the surface of the lungs slightly elevated variable-sized nodules ranging from 1.5 to 2 cm in diameter were observed. After cutting the surfaces revealed the presence of grayish exudates. In two sheep 2-3 cm pearly-white, hard nodules elevated from the surface of the parenchyma were observed particularly in diaphragmatic lobes along with congestion in rest of the portions of the lungs (Figure-2). Clinical history of the PCR positive lambs revealed symptoms of pneumonia. Grossly, the lungs of these lambs showed lesions of pneumonia unrelated to the OPA.

### Histopathology

**Figure 3:**
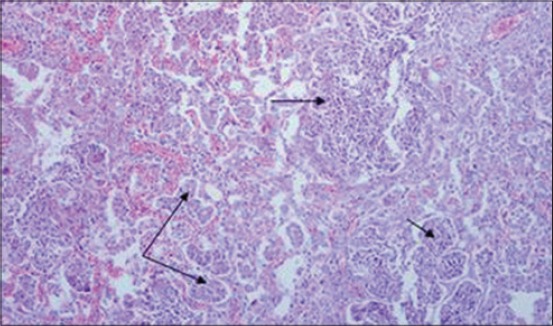
Lungs of ovine pulmonary adenocarcinoma polymerase chain reaction positive sheep showing proliferative changes in alveolar epithelial cells forming papillomatous projections into the alveoli (arrow) (H and E, 100×).

**Figure 4:**
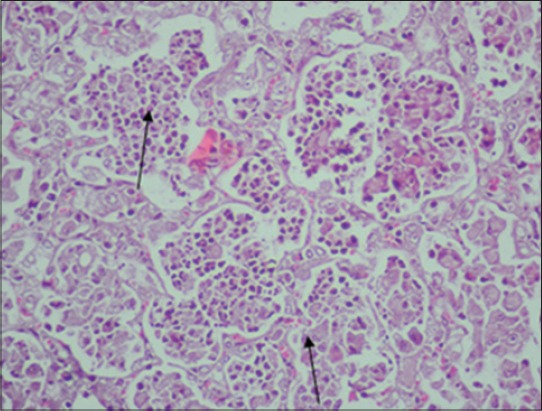
Alveolar epithelial cell proliferation and lymphocyte infiltration in the lungs of ovine pulmonary adenocarcinoma affected sheep (arrow) (H and E, 400×).

**Figure 5:**
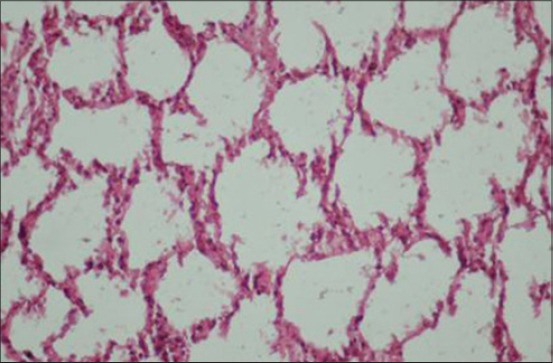
Lungs of polymerase chain reaction negative sheep for ovine pulmonary adenocarcinoma (negative control) (H and E, 400×).

Histological examination of lungs of adult PCR positive sheep revealed the characteristic proliferative changes in the alveoli. The alveoli revealed papillomatous proliferation forming well-marked papillae with distinct connective tissue cores giving adenomatous appearance. These papillomatous ingrowths partially or completely obstructed the alveolar lumen with projections into the alveoli (Figure-3). The bronchial and bronchiolar lining cells also revealed hyperplastic changes and rarely formed papillary projections which obstructed the bronchiolar lumen partially or completely. There was infiltration of numerous macrophages in the lumen and in the vicinity of proliferated alveoli was seen. In some of the lungs, interstitial spaces of the alveoli were found to be thickened with lymphocytes and plasma cells (Figure-4). A similar infiltration of mononuclear cells was also evident in peribronchial, peribronchiolar, and perivascular areas. In addition, the neutrophilic aggregates were observed in the lumen of several alveoli. The lungs of OPA negative control sheep did not reveal any histological alteration (Figure-5).

## Discussion

OPA is a contagious viral disease of sheep that results in pulmonary neoplasia mainly in sheep and rarely in goats. The economic losses can be statistically significant up to 80% of the flock on first exposure to the infection and continuing losses may occur up to 20% each year in some flocks [21]. Eradication of the disease from a flock is difficult because no diagnostic test can detect animals in the preclinical stage. In this study, the prevalence of OPA was observed in 8% of sheep naturally died due to pulmonary affections.

The gross lesions observed in the lungs of adult sheep and microscopic proliferative changes observed in the alveolar and bronchiolar epithelium were consistent with the observations of the previous OPA studies [2,9,22]. There was infiltration of numerous macrophages in the lumen and in the vicinity of proliferated alveoli as previously reported by various workers in both natural and experimentally induced OPA [23,24]. The pathogenic role of macrophages in OPA has not been fully demonstrated, however, it was suggested that these cells may be useful in clearance of excess surfactant secreted by the neoplastic type II pneumocytes. It was also demonstrated that the tumor cells secrete a chemotactic factor that might be responsible for the recruitment of macrophages [25]. Infiltration of the inflammatory cells particularly neutrophils in the alveoli of affected lungs in present report may reflect secondary bacterial infection [24,26]. Further, OPA affected sheep lack circulating anti-JSRV antibodies and have an increased susceptibility to secondary bacterial infection, indicate that OPA affected sheep are immunocompromised [27].

Diagnostic accuracy of JSRV PCR using field data from 125 Scottish sheep flocks was studied by Lewis *et al*. [28] using Primers P1 and P2 spanned a 229-bp region internal to the gag gene (position 1598 to 1826 of JSRV). Each blood sample of sheep was subsequently tested for the presence of JSRV proviral DNA using a hemi-nested PCR and the second round was a Taqman PCR using the carboxyfluorescein (6-fluorescein amidite; FAM) labeled probe. It was found the PCR had a high true (analytical) sensitivity and that the low observed (diagnostic) sensitivity in individual samples was due to low concentrations of target DNA in the blood of clinically healthy animals. In this study, of six PCR positive sheep, two were 3-6 months of age and did not show clinical signs/gross lesions suggestive of OPA. Histologically, lungs of these sheep showed pneumonic lesions without evidence of OPA. The positivity of these sheep in early age reflects the sensitivity of the PCR assay used in this study. The high sensitivity for detection of OPA with improved PCR techniques has been previously reported in lymphoreticular system and peripheral blood mononuclear cells before the onset of neoplasia in experimentally inoculated lambs [27], during the pre-clinical period of the natural disease in the sheep flocks and in the in-contact sheep with no evidence of pulmonary neoplasia [29]. Our study was aimed to detect the existence of OPA in the naturally died sheep due to pneumonia in the farm under study. The gross and histopathological studies carried out to evaluate OPA lesions in the lungs. We optimized the PCR assay using published primer sequences specific for JSRV and used as a secondary confirmation test. The sensitivity and specificity of assay with these primer sequences have already been reported by previous workers [4]. The absence of gross and pathological lesions of OPA in the lungs may be due to non-development of tumor as the OPA lesions are thought to be age-dependent and clinical signs could be seen in those animals that have developed tumors [30]. In contrast to other ovine retroviral infections like Maedi-Visna, there is no known specific antibody response to infection by JSRV [18]. Therefore, there is no diagnostic serological test available [4]. Other diagnostic aids such as ultrasonography, lung biopsy, cytological and biochemical analysis of bronchoalveolar lavage, lacks sufficient sensitivity and specificity for diagnosis of early stages of the disease [3].

## Conclusions

Based on the pathological investigation and PCR results, this study concludes that the OPA was prevalent in the sheep farm under study. PCR assay used on lung tissues would be helpful in the screening of preclinical and clinical cases of OPA on postmortem in sheep. However, there is need to develop PCR assays in clinical samples such as bronchoalveolar lavage, blood, colostrum, and milk in the OPA affected sheep flocks in India for early clinical diagnosis of OPA so as to reduce the incidence of the disease.

## Authors’ Contributions

GGS and BNT designed the study. GGS, JK, and RK conducted the study, the study progress monitored by BNT. GGS prepared the original draft of the manuscript. All authors read, revised, and approved the final manuscript.
